# Central Asian Post-Soviet health systems in transition: has different aid engagement produced different outcomes?

**DOI:** 10.3402/gha.v7.24978

**Published:** 2014-09-16

**Authors:** Anar Ulikpan, Tolib Mirzoev, Eliana Jimenez, Asmat Malik, Peter S. Hill

**Affiliations:** 1 School of Population Health, The University of Queensland, Herston, QLD, Australia; 2 Nuffield Centre for International Health & Development, Leeds Institute of Health Sciences, University of Leeds, Leeds, UK; 3 Integrated Health Services, Islamabad, Pakistan

**Keywords:** aid effectiveness, health sector, Central Asian Post-Soviet countries, donor aid

## Abstract

**Background:**

The collapse of the Soviet Union in 1991 resulted in a transition from centrally planned socialist systems to largely free-market systems for post-Soviet states. The health systems of Central Asian Post-Soviet (CAPS) countries (Kyrgyzstan, Mongolia, Tajikistan, Turkmenistan, and Uzbekistan) have undergone a profound revolution. External development partners have been crucial to this reorientation through financial and technical support, though both relationships and outcomes have varied. This research provides a comparative review of the development assistance provided in the health systems of CAPS countries and proposes future policy options to improve the effectiveness of development.

**Design:**

Extensive documentary review was conducted using Pubmed, Medline/Ovid, Scopus, and Google scholar search engines, local websites, donor reports, and grey literature. The review was supplemented by key informant interviews and participant observation.

**Findings:**

The collapse of the Soviet dominance of the region brought many health system challenges. Donors have played an essential role in the reform of health systems. However, as new aid beneficiaries, neither CAPS countries’ governments nor the donors had the experience of development collaboration in this context.

The scale of development assistance for health in CAPS countries has been limited compared to other countries with similar income, partly due to their limited history with the donor community, lack of experience in managing donors, and a limited history of transparency in international dealings. Despite commonalities at the start, two distinctive trajectories formed in CAPS countries, due to their differing politics and governance context.

**Conclusions:**

The influence of donors, both financially and technically, remains crucial to health sector reform, despite their relatively small contribution to overall health budgets. Kyrgyzstan, Mongolia, and Tajikistan have demonstrated more effective development cooperation and improved health outcomes; arguably, Uzbekistan and Turkmenistan have made slower progress in their health and socio-economic indices because of their resistance to open and accountable development relationships.

In 1991, 2 years after the fall of the Berlin Wall, the collapse of the Soviet Union radically transformed the political and the economic context in the Central Asian states previously dependent on the Soviet Union's economy: Kazakhstan, Kyrgyzstan, Tajikistan, Turkmenistan, and Uzbekistan. Mongolia was similarly affected. Although not a member of the Post-Soviet Commonwealth of Independent States, it shares the same socio-political and economic history. The Union of Soviet Socialist Republics (USSR) provided one-third of Mongolia's gross domestic product (GDP) prior to the collapse, and dominated Mongolian political decision making (1).

All former Soviet countries were hit hard; these countries experienced declines between 33 and 60% of GDP from 1989 levels reaching their lowest point in 1995–96. Uzbekistan had the lowest fall of 15% in GDP (2, 3). Public health spending as a proportion of GDP declined sharply for most Central Asian Post-Soviet (CAPS) countries, falling to between 2.1 and 3.5% of GDP during 1991–1996. Tajikistan experienced the most precipitous decline in GDP because of its civil war, with health funding dropping from 6% of GDP in 1991 to only 1.1% in 1996 (4). Mongolia's decline in health expenditure was less dramatic, falling from 5.7 to 4.4% of GDP in the same period (5), cushioned by inputs from external donors, who contributed nearly one-third of the GDP at that time (6).

Under such constrained circumstances, the provision of health services became challenging. Although the CAPS countries mirrored each other in many aspects of socio-economic development, institutional arrangements of health, education, and social-welfare systems (4, 7), early differences among these newly post-Soviet states were becoming apparent, especially in their relationships with donors. For all CAPS countries, the economic vacuum left by the withdrawal of Soviet support needed to be urgently replaced. Donor relationships expanded to include Western European and Nordic countries, Japan, the United States, and South Korea, and cooperation with the major multilateral agencies and development banks was established (4, 8, 9).

One of the factors that distinguished the different trajectories of health outcomes in these states since 1991 appears to be their engagement with these new donors. After 70 years of Soviet domination, CAPS countries did not have the experience to enter into collaboration and negotiation with multiple donors, and the capacity to do so was inextricably linked to their political transitions (4). Donors also needed time and expertise to adapt their development assistance portfolios to this new development challenge, engaging these hierarchical and less-transparent post-Soviet states. Early donor interventions included ad-hoc relief aid, humanitarian assistance, and small pilot projects (4, 10, 11). These multiple and uncoordinated projects increasingly created an administrative burden on the fledgling health ministries, causing an acute need for coordination (12–14) . Limited local capacity resulted in international partners trying to drive health sector reforms and manage development coordination (15), though this was constrained by their differing agendas, incompatible financial and reporting forms and procedures. Without effective coordination, donors competed between themselves and with recipient governments over the human and financial resources (16, 17). The international advocacy for local ownership, harmonisation, and alignment that would be encoded in the Paris Principles on Aid Effectiveness (18) contrasted with the deeply centralised Semashko health systems model inherited from the Soviet Union, with its bureaucratic inertia, centralised management, and lack of transparency and accountability. The Semashko model had been established in the 1920s and was in operation throughout the Soviet Union until the early 1990s (19). ‘The model was characterized by its centralised planning and administration, government financing and provision of services through publicly owned health care providers, which were universally accessible and free at the point of delivery’ (19), p. 421). While the model ensured population coverage with basic health care, issues of efficiency and quality of care were not addressed. The model was largely curative in its focus with massive infrastructure costs that made it too ‘inappropriate and inefficient’ to meet the changing health needs of the population or the market economies of these emerging post-Soviet democracies (9, 12, 20–22) .

Despite huge social and political changes, and the significant accompanying reforms in the health sector, research in these post-Soviet states has been neglected. This is reflected in the paucity of recent publications on health systems in these states, and the limited access of the international community to Russian language publications (15, 23, 24). In an analysis that compares publications to population size as a gauge of significance, CAPS countries’ health sector have been ranked as ‘least studied’ with only 0.16–1.71 publications per 100,000 population (24). We found no studies comparing the influence of development assistance on CAPS countries possibly because of these countries’ less aid experience. This research provides an analysis of the transition period from central control to democratic economies in selected low middle-income CAPS countries (Kyrgyzstan, Tajikistan, Turkmenistan, Uzbekistan, and Mongolia), examining the role of development assistance in health since the Soviet Union collapse.

## Methods

A multi-case study design (25) was used to examine development assistance in health in CAPS countries in relation to their broader socio-economic and political contexts. Three research methods were used: 1) extensive document review 2) key stakeholder interviews for in-depth understanding of both donors’ and governments position in aid coordination and its effectiveness; 3) participant observation based on the two authors’ (AU and TM) 10–15 years of experiences of working in health system reform projects in Mongolia and Tajikistan, respectively. The criteria for selection included their socio-political history as post-Soviet states, and shared regional Central Asian status. The comparability of their socio-economic and health indicators, disease burdens, types of health system, and political regimes at the point of the collapse of the Soviet Union provides a common base from which to observe changes in their socio-economic and political development, and their engagement with development assistance. Kazakhstan has been excluded from the analysis because of its prospering economy, limited requirement for development assistance, and early transition to upper-middle-income status.

### Document review

Pubmed, Medline/Ovid Scopus and Google scholar search engines were used for peer-reviewed journals in English, Russian, and Mongolian using a combination of key words: ‘development assistance’, ‘external aid’, ‘donor assistance’, ‘aid effectiveness’, ‘sector-wide approach’, and ‘health sector’. The terms ‘Soviet countries’, ‘Central Asian countries’, and names of each country acted as a further filter.

Websites of international development institutions, public health research institutes, and ministries of health of each study country were also explored to examine various indicators, including those related to health status and other factors directly relevant to aid effectiveness and donor coordination such as the control of corruption, ease of doing business index, and geopolitical and economic influence. The country health reports and health sector strategies that documented these countries’ socio-economic change and health sector performance over the past two decades are mostly in Russian and Mongolian, and were accessed by AU, who is fluent in Russian, and a native Mongolian speaker, and TM, a native Russian speaker. In total, over 100 references published in English, Russian, and Mongolian were reviewed; out of these, 92 references were cited in the final review, comprising 78 in English, 7 in Russian, 7 in Mongolian. Data analysis and the writing of the manuscript were done in English.

### Interviews

In-depth interviews were conducted with 11 purposively selected key informants with experience in Kyrgyzstan, Mongolia, and Uzbekistan. Informants were selected for their depth of experience across CAPS country health sector reform processes. This complemented the authors’ direct experience of Tajikistan (TM) and Mongolia (AU, PSH). A profile of key informant experience is provided in Annex 1.

All the interviews were face-to-face, with informed consent, and held in Ulaanbaatar, Mongolia, with two interviews conducted in Mongolian and the remainder in English.

### Participant observation

Two authors (AU and TM) participated at the policy making level in the health sectors of Tajikistan and Mongolia from 1998 to 2011, providing direct experience of the changes that occurred during the transition period. Their involvement in donor-funded projects in health allowed the observation of project implementation challenges and the changes that occurred over time. Their engagement with government and donor projects, and their roles as both participants as well as researchers, has given them both ‘insider’ and ‘outsider’ perspectives, depending on the context (26).

### Data analysis

Comparison of the quantum of development assistance in CAPS countries was undertaken using the proportion of aid compared to the burden of disease and the amount of Overseas Development Assistance (ODA) received per capita. Other comparative analyses included key socio-economic and health indicators; Paris Declaration indicators for aid effectiveness (Table 4); key informants’ interviews on CAPS countries’ health sector and role of the external aid and the use of the framework to explore evolutionary stages for donor coordination (Table 5).

### Limitations

The limited published literature – even including Russian language sources – has constrained the depth of cross-country comparisons. Moreover, data for development assistance for health in CAPS countries are patchy and limited. Nevertheless, this has been compensated through triangulation with interviews and other sources of information (country reports, consultants’ reviews, OECD reports, donors’ evaluation reports, and government documents).

## Findings and discussion

We begin with an overview of the *socio-economic and health status* since collapse of the Soviet Union. The *significance of development assistance* and *aid delivery approaches* is critically reviewed. We then identify the *main actors* involved and those *factors that directly affect aid effectiveness* are explored along with these countries’ progress on *Paris Declaration indicators*. Finally, *aid coordination mechanism* operations are explored to identify the maturity of the aid coordination in these contexts.

### How has socio-economic and health status changed since the Soviet Union collapse?

Poverty has remained a persistent issue among the CAPS countries. As late as 2010, 46.9% of the population in Tajikistan were living below the national poverty line; in Kyrgyzstan, 31.7% fell under the poverty line (27). The IMF Country Report for Uzbekistan reports a poverty rate decline from 26% in 2005 to 20% in 2010 (28), and in Mongolia, despite its growing economy, the poverty rate remained at 39.2% in 2010, marginally increasing from 36.3% in 1995 (27). In Turkmenistan, information is limited, though a study undertaken by USAID indicated that in 2003, 58% of the population in Turkmenistan lived below the national poverty line (29). Compared with other higher MICs, these levels point to high internal inequality and consistent with the view that in the context of high inequality, democratisation is less likely (30) and Turkmenistan is the least democratised country among the CAPS countries.

Health and socio-economic indicators declined sharply after collapse of the Soviet Union due to chronic funding shortage and inefficient management (19, 31, 32). Economic growth during 1990–2005 was unstable, with the substantial downturn causing social upheaval and lowering health indicators, and unemployment and poverty rates increased dramatically. External aid was urgently needed but did not immediately flow as needed during the early 90s (32), except in the case of Mongolia (6). Though Mongolia is currently a middle-income country, in 1999 it was one of the four most aid-dependent countries, with aid constituting more than 25% of GNI (6).

**Fig. 1:**
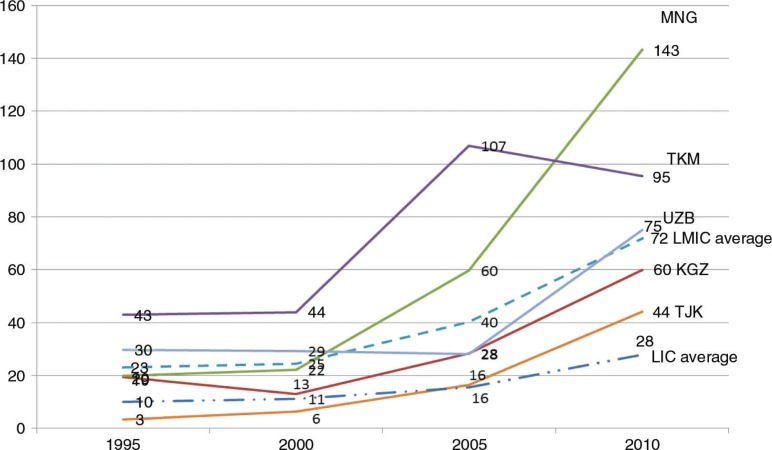
Health expenditure per capita (in US$) in selected CAPS countries and averages of low-income and lower middle-income countries. Source: World Bank databank (27).

The overall pattern in health expenditure in four of the five countries has been similar, increasing slowly between 1995 and 2010, with the exception of Turkmenistan (Fig. 1). Tajikistan has the lowest health expenditure amongst the CAPS countries despite its high demand for investments in health (9). Both domestic and external resources need an increase. Nevertheless, Tajikistan made the highest progress in reducing maternal mortality from 120 to 65 during 2000–2010 (27, 33) even though the country spending on health is about half Turkmenistan's spending. In contrast, despite its highest per capita health expenditure during 1995–2005, Turkmenistan has the poorest health indicators among CAPS countries.

Tables 1 and 2 compare key health and socio-economic indicators of these countries after two decades of the transition, comparing them with overall averages for low- and middle-income countries.

**Table 1:** Central Asian Post-Soviet countries: GDP, ODA, and selected health indicators

Country	GDP per capita US$ (2011)	Net ODA received percentage of GNI (2010)	External resources for health (% of THE on health) (2010)	Health expenditure total (% of GDP) (2010)	Life expectancy at birth (2010)	Maternal mortality ratio per 100,000 (modelled estimate 2010)	Under 5 mortality per 1,000 (2010)
Tajikistan (LIC)	$935	7.7%	6.1%	6%	67	65	63
Kyrgyzstan (LIC)	$1,075	8.7%	12.8%	6.2%	69	71	38
LIC average^a^	$534	10%	25.8%	5.3%	59	410	109
Uzbekistan (LMIC)	$1,546	0.6%	0.9%	5.8%	68	28	52
Mongolia (LMIC)	$3,056	5.4%	3.9%	5.4%	68	63	32
Turkmenistan (UMIC)	$4,722	0.2%	0.3%	**2.5%**	**65**	**67**	**56**
MIC average^b^	$3,732	–	0.6%	5.7%	69	190	51

Source: World Bank databank (27).

a Low-income country (LIC) average.

b Middle-income country (MIC) average.

UMIC=upper middle-income country; LMIC=lower middle-income country.

THE=Total Health Expenditure.

Bold values indicate low performance compared to country economic status.

**Table 2:** CAPS countries’ key socio-economic and health systems indicators

Country	Total population (2012, WB)	Unemployment rate % total (2010, WB)	Percentage of population living below the national poverty line (2010, WB)	Literacy rate (2010) WHO	DALYs per 1,000 population (total, all causes, all ages 2010, IHME)	Health workforce (2011) per 10,000* WHO
Tajikistan (LIC)	8,000,900	11.6	46.9 (2010)	99.7	352	Physician: 18 Nurse: 44
Kyrgyzstan (LIC)	5,474,000	8.6	31.7	99.2	386	Physician: 19.6 Nurse: 61.2
LIC average**	N/A	5	49.1	N/A	599 (2012)	Physician: 2 Nurse: 6
Uzbekistan (LMIC)	28,541,000	11.4	20	99.4	336	Physician: 25.6 Nurse: 111.5
Mongolia (LMIC)	2,796,000	6.5	39.2	97.4	400	Physician: 27.6 Nurse: 35
Turkmenistan (UMIC)	5,173,000	11.4	58.2 (29)	99.6 (2010)	311	Physician: 23.9 Nurse: 44.2
LMIC/UMIC average**	N/A	5 (LMIC) 6 (UMIC)	28.2 (LMIC) 8.7 (UMIC)	N/A	454/291(2012)	LMIC: Physician: 8 Nurse: 18 UMIC: Physician: 18/10,000 Nurse: 27/10,000

Sources: World Bank and WHO (27, 36).

* WHO estimates that countries with fewer than 23 physicians, nurses, and midwives per 10,000 population generally fail to achieve adequate coverage rates for selected primary health care interventions.

** LIC=low-income country

** LMIC=lower middle-income country; UMIC**=upper middle-income country.

Overall, health indicators in CAPS countries compare positively with the averages for lower middle-income countries (LMICs) (Table 1), arguably as a result of the extensive health and education infrastructure and adequate human resources established during the socialist period. However, the data accuracy and reliability of post-Soviet information systems has often been questioned (9, 20, 22). Key health indicators have improved in all countries – though at different paces – from 2000 to 2010, with highest progress in the declining Maternal Mortality Rate (MMR) and Under 5 Mortality Rate (U5MR) observed in Tajikistan and Mongolia (27). This has been associated in some analyses with the increased donor aid in these areas and aid coordination efforts (9, 34). However, CAPS countries face major inequalities: the poor and rural populations are most affected (20, 22, 34). Turkmenistan has the second highest MMR and U5MR in this cluster, despite its recent move to upper middle-income country status: economic growth has not significantly benefitted population health. The Government of Turkmenistan has allocated the lowest percentage of GDP to health (2.5%), among the CAPS countries. Other CAPS countries included in the study spent 5.4–6.2% of GDP on health, which meets the WHO Commission on Macroeconomics and Health recommendation (35).

Their similar political and economic histories mean that socio-economic indicators (Table 2) of these CAPS countries do not differ much. Despite the challenges faced in the acute disruptions to their economies, none of the CAPS countries are failed states; in the after-shock, their government functions and services were severely downscaled but still continued (37–39) . Unemployment is a relatively new phenomenon; in the Soviet Union, state enterprise guaranteed employment. Unemployment rose after the collapse of the Soviet Union with the closure of many factories and some health and education facilities with the cessation of Soviet funding (40–42) . In the past decade, the development of the private sector has contributed to an increase in employment opportunities; nevertheless unemployment is still quite higher in these countries compared to the LMIC averages (27, 43). Poverty is also disproportionally high in comparison with the unemployment rate. On the positive note, a literacy rate is consistently high in all these countries as a result of universal education policy during the socialist regime.

Among the CAPS countries, DALYs per 1,000 population are lower than LMIC averages, except Turkmenistan, which has slightly higher DALYs compared with upper middle-income country (UMIC) averages. The ‘double-burden’ of disease is present in all these countries with increasing dominance of non-communicable diseases over the last two decades and persisting communicable disease burdens (44). These post-Semashko model health systems continue to maintain higher numbers in their health workforce compared with other LMICs, and cost-effectiveness and quality of services continues to be a concern (15, 32, 45).

### How significant has development assistance been?

The amount of ODA received by each of the CAPS countries varies considerably. Compared to other countries with similar levels of child mortality, life expectancy and health expenditure, all former Soviet countries receive very low development assistance for their health sectors (46). Kyrgyzstan, Tajikistan, and Mongolia's aid per capita is relatively high, yet does not yield corresponding positive health outcomes, needing more effective aid coordination. Uzbekistan and Turkmenistan receive disproportionally low amount of aid compared to their overall burden of disease (27, 47), falling into the Very Low Aid Countries category despite their increasing poverty and evident needs for better performance (48). The remaining CAPS countries meet the Middle-Aid Countries criteria, yet the average external resources for health in these countries is lower than in other countries with similar levels of health indicators (46). The reasons for this are often explained by their political history, less openness and transparency in their organisational management culture. An international consultant reports:

Soviet style management by its nature does not promote information sharing, openness and transparency. When I started my work first time in Mongolia, then in Kyrgyzstan, the challenges I faced were very similar. Officers are afraid to provide health information and especially if it was about what is not working properly, they are extra cautious. It is definitely inherited from their long-standing culture of punitive management and it works against their effective collaboration with international partners.

**Fig. 2:**
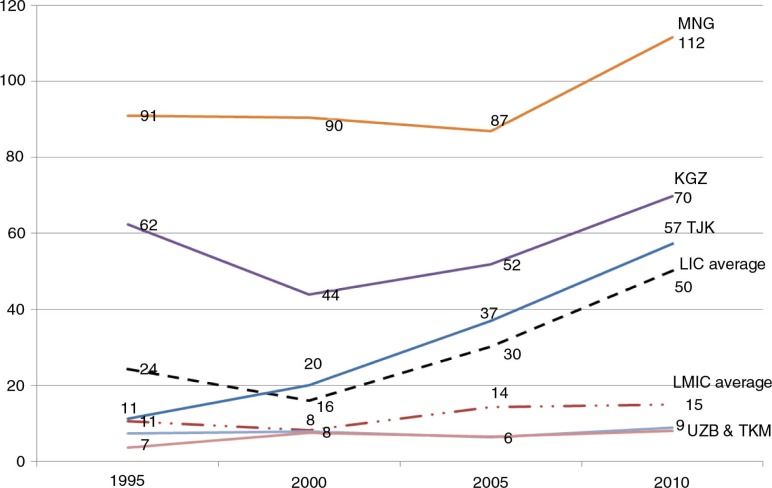
Net ODA received per capita (in US$) in selected CAPS countries and averages of low-income and lower middle-income countries. Source: World Bank databank (27).

Mongolia receives the highest ODA per capita amongst CAPS countries (Fig. 2), much higher than highly aid-dependent countries such as Cambodia and Mozambique with US$37.26 and US$76.82 ODA per capita, respectively (27). Tajikistan and Kyrgyzstan have made efforts to improve their aid coordination in the last few years, and consequently, they are attracting more aid since 2000. The low ODA per capita of US$6.9 USD in Turkmenistan and Uzbekistan is likely to be because of their bureaucratic governance and less open relationship with potential donors (49) rather than their being considered as self-sufficient, since both countries have significant levels of poverty (29).

While external assistance for health is not as high in monetary terms compared to other developing countries, the health reform processes in CAPS countries are highly dependent on the international technical assistance.

### How has aid been delivered?

Globally, aid modalities have changed from mostly project-based vertical approaches to sector-wide horizontal approaches and budgetary support. These changes have been observed in three CAPS countries: Kyrgyzstan, Mongolia, and Tajikistan. The Paris Declaration principles have played a role in improving development aid practice in both donor and recipient partners.

### Aid modalities

During early 2000, most of the aid provided to CAPS countries was delivered in project-aid form, important in salvaging failing health systems in the early transition, but less useful in terms of sustainable health systems strengthening. The project aid allowed rapid responses by donors to targeted, disease-specific issues – often with limited consultation with local policymakers. These multiple, short-term, ‘vertical’ disease-specific interventions, while often meeting their immediate objectives, increasingly created fragmentation, conflicting priorities, and additional administrative burdens for local authorities (46). As global trends moved towards more effective ways of delivering aid, more transparent and mutually accountable approaches such as programmatic aid and direct budgetary assistance have been strongly promoted by donors and welcomed in Tajikistan, Kyrgyzstan, and Mongolia. In fact, while these new aid relationships may not have had precedents for either donors or recipients, they have offered fresh opportunities for both to redefine their modes of operation. This has been less the case in Uzbekistan and Turkmenistan, where ‘vertical’ disease control projects, often led by UN agencies and global health initiatives continue to dominate development assistance (29, 50).

With the desire to coordinate development assistance and reduce the duplication and inefficiencies of project-aid, sector-wide approaches (SWAps) have been introduced in many LMICs. SWAps promote country leadership and effective collaboration of partners to support a single sector policy envelope, and prioritise the strengthening of local capacity for managing programme implementation, monitoring, and evaluation (51, 52). Tajikistan, Kyrgyzstan, and Mongolia are moving towards a SWAp with varied progress (9, 12, 22, 53). Because of their long-standing culture of centralised administration, the SWAp, with its re-centralising tendency may be well-suited to post-Soviet countries. Informants saw significant advantages to SWAps where CAPS countries had already achieved middle-income status:

These countries should use their relative advantages such as less-dependency, negotiation power and political stability for the benefit of aid effectiveness. These advantages provide favourable environment for an effective SWAp, which is planned to be implemented some of these contexts. (UNICEF officer)

Tajikistan has not yet shown major progress in any of the key elements of SWAp (54) whereas Kyrgyzstan is progressing well, with strong donor coordination through this first health SWAp in a post-Soviet country (22, 55, 56), though parallel projects funded by various agencies also co-exist (22). In Kyrgyzstan, government allocations to health increased steadily since the government's explicit commitment to a SWAp (27, 57). European Union donors have played a key role in the Kyrgyz health sector: since 2006, funds from key donors have been allocated within the framework of a SWAp. The National Health Reform Programme in Kyrgyzstan has recently been evaluated using the IHP+ framework for their Joint Assessment of National Strategies, facilitating donor collaborations.

The Mongolian health sector has seen the development of a clear national health plan, increased ownership and willingness to coordinate partners under this plan, providing a solid basis for a SWAp (58, 59). However, the SWAp momentum has not been maintained. The frequent turnover of senior Ministry of Health (MoH) staff has definitely slowed down the process, and donors’ reluctance or uncertainty about government procedures has tempered their enthusiasm (60). Interviewees from bilateral donors indicated that unless the government improves the transparency and accountability in its governance and financial management procedures, they will find it hard to commit to a SWAp despite the increasing government leadership and capacity over the process.

Budgetary support has increased in Mongolia from 29% in 2006 to 32% in 2010 (3, 61); in Kyrgyzstan, the baseline increased from 12 to 21% over the same time period, with Tajikistan reporting 8% budgetary support in 2010 (9, 55, 56). All three countries are still far from the OECD's 66% target by 2010. There were no reports regarding direct budgetary support for Uzbekistan and Turkmenistan.

### Who are the donors in health?

CAPS countries have relatively few donors in health [Table 3 (9, 12, 20, 22, 62, 63)] but still maintain their need for effective aid coordination (62, 64). Development assistance is said to be fragmented when there are more than 15 donors, between them providing less than 10% of the country's programmable aid (65). The fragmentation of health aid causes burden to these health systems in transition and interferes with cohesive health policy process (12, 60, 62, 66). Three CAPS countries fall into this fragmented category: Tajikistan, Kyrgyzstan, and Mongolia. Table 3 shows number and type of heath donors involved in CAPS countries.

**Table 3:** External development actors in health in CAPSC (see Annex 2 for abbreviations)

		Type of external actors in health (2000–present)				
		
Countries (ordered by income categories from the lowest to the highest)	Number of actors active in health (approximate estimate since 2000)	Bilateral agencies (ordered by relative size of ODA contribution)	Multilateral agencies			
		
			UN agencies	Development agencies	International NGOs	Global health initiatives
Tajikistan	53 partners funded and implemented 97 projects (as of 2006) (62)	- DFID - European Union - SDC - SIDA - GTZ - DANIDA - USAID - CIDA - Italy - The Netherlands	- WHO - UNICEF - UNAIDS	- World Bank - ADB - Agha Khan Foundation European Bank for Reconstruction and Development - German Development Bank - Islamic development Bank	- Soros Foundation - ACT Central Asia - OXFAM	- Global Fund - GAVI
Kyrgyzstan	10–20 (not more than 10 in a given year)	- DFID - SDC - SIDA - USAID - GTZ - JICA	- WHO - UNFPA - UNICEF - UNAIDS	- World Bank - German Development Bank - Agha Khan Foundation	- MSF - Red Cross - Soros Foundation (SF)	- Global Fund - GAVI
Uzbekistan	Less than 10 since 1990	- USAID - GTZ - JICA	- WHO - UNICEF - UNFPA	- World Bank - ADB	N/A	- Global Fund - GAVI
Mongolia	10–20 (had highest number of donors in the region in early 2000; past 5 years not more than 10 in a given year)	- USAID - Luxembourg - GTZ - Belgium - AusAid - Switzerland - Italy - JICA	- WHO - UNICEF - UNFPA - UNAIDS	- ADB	- Open Society Forum (former SF) - World Vision - Norwegian Lutheran Mission (NLM) - VSO	- Global Fund, - GAVI
Turkmenistan	Less than 10 since 1990	- USAID	- WHO - UNFPA - UNICEF	N/A	N/A	- Global Fund (TB only) - GAVI

Sources: (9, 12, 20, 22, 62, 63) official websites of JICA, GIZ, DFID, World Bank, ADB, WHO, UN agencies, Agha Khan Foundation, World Vision, Soros Foundation, GF, GAVI.

Key informants were also concerned about some bilateral agencies’ unwillingness to commit to long-term capacity building initiatives such as SWAp. With the exception of some European donors and the German GIZ, bilateral agencies’ engagement in CAPS countries has been ad-hoc and short-term, without the necessary long-term development interest (49). Even where SWAps have been established, these agencies have continued providing project aid in parallel to the SWAp (55, 63, 66, 67). In the case of Mongolia, key supporters of the health SWAp have been ADB, UNFPA, and WHO. Development banks’ efforts have been more influential on policy directions in health. Non-Government Organisations (NGOs) in health are a new phenomenon in Central Asia, and have played a less significant role.

There are two divergent patterns for development assistance in CAPS countries. Tajikistan, Kyrgyzstan, and Mongolia have the higher numbers of various partners (9, 12, 22). Mongolia has been most successful in opening up its relationship with the United States, Japan and, the Western countries because of its earlier concrete political steps to democratisation (1, 68). In 1991, ODA reached its peak amounting to 165% of GDP in Mongolia, more than half of it coming from Japan, the United States, and Germany in the form of grants and soft loans to support infrastructure and social sector, including health (61, 69). Since then, ODA had been gradually decreased down to 18 and 4% of GDP in 2001 and 2009, respectively, in response to the mineral-resource-driven economic growth (61, 69). Nevertheless, the early fuelling of ODA had greatly supported Mongolia to overcome transition challenges.

The spread of aid actors in Uzbekistan and Turkmenistan has been limited. The limited involvement of aid actors in the two countries is, in part, due to their intransigent political regimes and neglect of human rights issues, as well as concerns about corruption (19, 29). The resistance to structural change and limited transparency has been a disincentive for development partners, though these are part of a more complex set of factors at play in development politics.

The geopolitical positioning of the CAPS countries between Russia, China, and India has raised concerns that external donors’ interests may be more geopolitical than developmental in motivation, and current political developments in former Soviet states underline this concern (49, 70). The landlocked position of CAPS countries, coupled with their rich mineral resources, make them attractive to the geopolitical interest (71–73) of both traditional and emerging donors. Russia, China and India, three of the BRICS nations have been active in business investment, but they have not contributed to development assistance in health in CAPS countries.

### What factors influence aid effectiveness?

Aid is effective only where there is adequate transparency and accountability (74, 75). The extent of democratic reform from a planned economy towards a market economy in post-Soviet countries appears to be one of the key determinants of the levels of external partners’ involvement (69, 76, 77), with the control of corruption, and the promotion of voice and accountability, linked to effective aid implementation as suggested in.

**Fig. 3:**
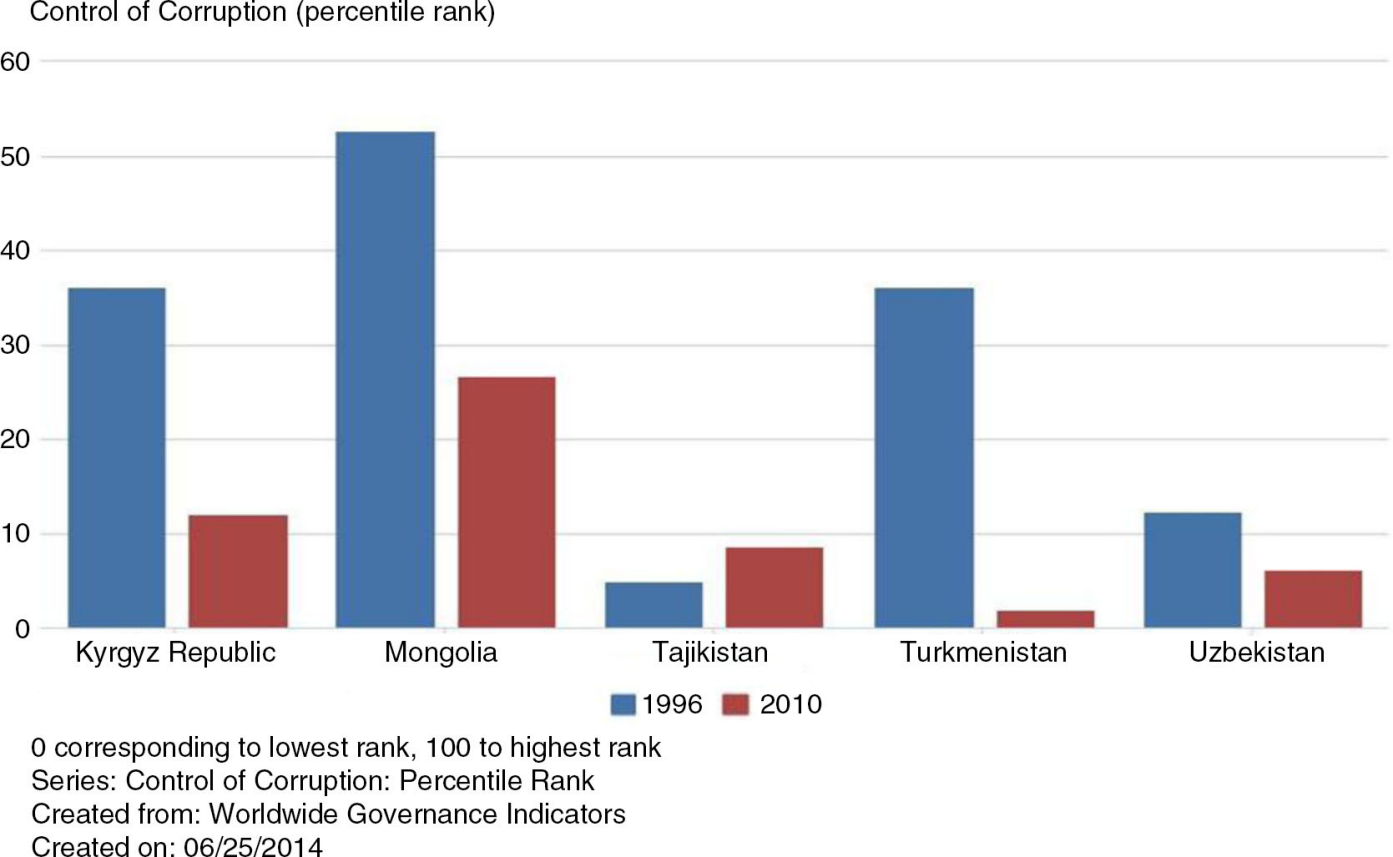
Control of corruption index percentile rank comparison of 1996 and 2010. Source: World Bank databank (27).

In each of the CAPS countries, the percentile ranking of the control of corruption (Fig. 3) has significantly fallen between 1996 and 2010, with the exception of Tajikistan. While Mongolia remains highest among the selected CAPS countries in terms of corruption control, by 2010 its ranking had fallen to almost half its ranking in 1996. Turkmenistan's ranking has dramatically lowered from 36 to 2, and is now the lowest in terms of corruption control, lower even than Uzbekistan.

**Fig. 4:**
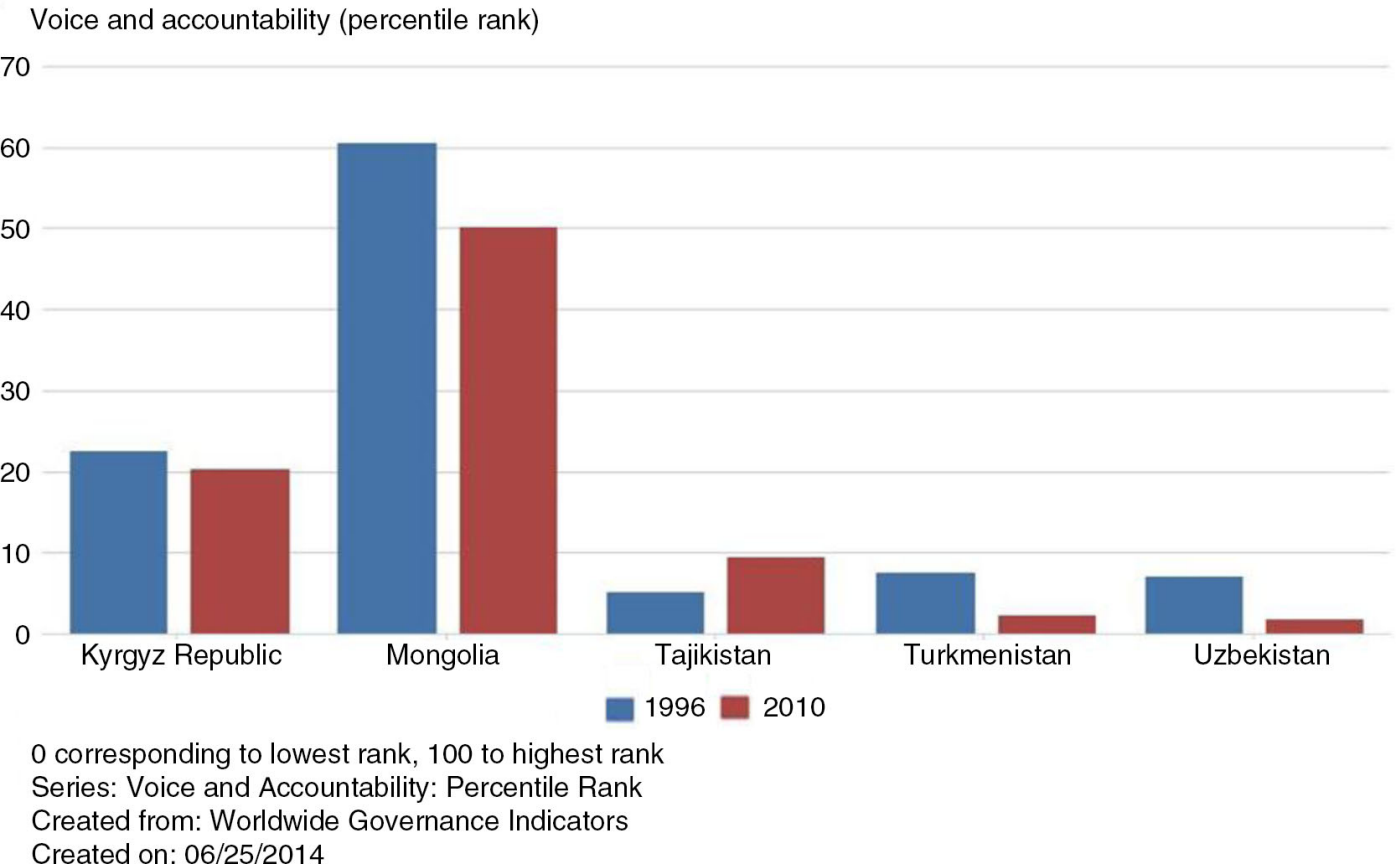
Voice and accountability index percentile rank comparison of 1996 and 2010. Source: World Bank databank (27).

A similar tendency has been observed in the voice and accountability indices (Fig. 4); all CAPS countries’ rankings were lowered, except Tajikistan. Turkmenistan and Uzbekistan's ranking has been persistently low among the selected CAPS countries, reinforcing the findings about the countries’ rather slow transition to democracy and openness.

**Fig. 5:**
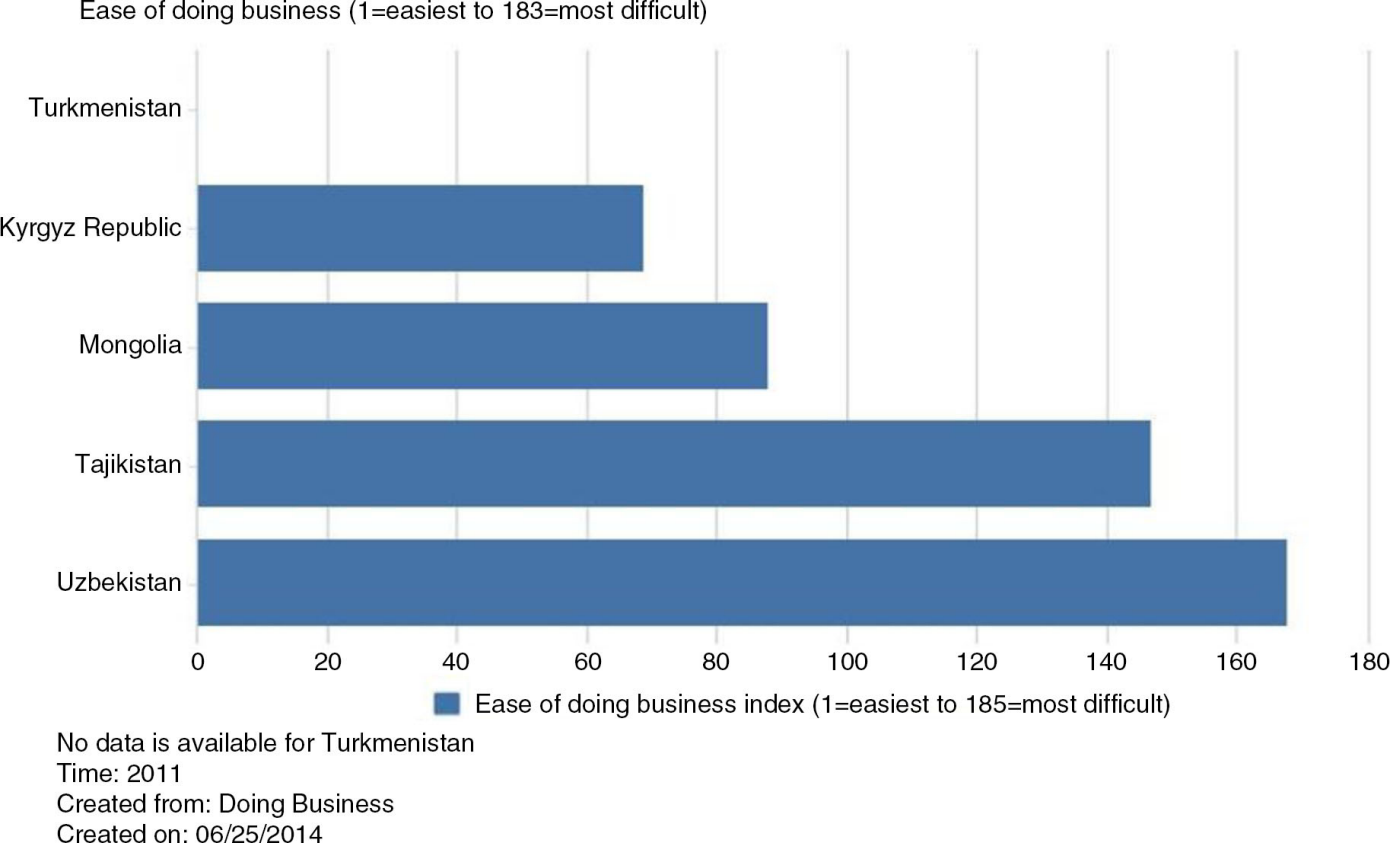
Ease of doing business index. Source: World Bank databank (27).

The Ease of Doing Business index (Fig. 5) also contributes to the assessment of the robustness of aid environment. In 2011, Kyrgyzstan ranked the easiest (at 69) among the group, whereas Uzbekistan's ranking was very low at 168, indicating the rather difficult environment for doing business in the country which further confirms its limited cooperation with donors. There was no indicator available for Turkmenistan, and no indicators for previous years were identified for the remaining countries.

It is widely acknowledged that quantifying aid effectiveness is challenging, and attributing its contribution to health system outcomes difficult to isolate from the complex of issues that impact broadly on health (78–80) . Arguably, the process of development assistance should point towards long-term change, and the OECD has devised indicators against which donor conformity with the Paris Declaration on Aid Effectiveness (Table 4) might be measured. Though these are not confined to the health sector, they reflect the extent to which donors prioritise government leadership and processes, harmonise approaches with each other and align activities with government policy.

**Table 4:** Country performances on the Paris Declaration Survey 2011

	Kyrgyzstan		Mongolia		Tajikistan	
	
Paris declaration survey indicators	2010 Target	Actual	2010 Target	Actual	2010 Target	Actual
Ownership^a^						
Operational development strategies	B or A	D	B or A	C	B or A	C
Alignment						
Reliable public financial management (PFM) systems^b^	3.5	3.5	4.5	4	3.5	3.5
Reliable procurement systems^c^	No target	N/A	No target	N/A	No target	N/A
Aid flows are aligned with national priorities (aid on budget)	85%	24%	85%	19%	85%	50%
Strengthen capacity by coordinated support	50%	81%	50%	81%	50%	83%
Use of country PFM systems	35%	32%	66%	27%	No target	31%
Use of country procurement systems	No target	28%	No target	21%	No target	30%
Strengthen capacity by avoiding parallel project implementation units (PIUs)	28	80	27	52	No target	15
Aid is more predictable	83%	44%	74%	30%	No target	91%
Aid is untied	More than 94%	77%	More than 86%	82%	More than 78%	66%
Harmonisation						
Use of common arrangements or procedures (programme-based approaches – PBAs)	66%	21%	66%	32%	66%	8%
Joint missions	40%	20%	40%	10%	40%	22%
Joint country analytic work	66%	22%	66%	21%	66%	50%
Managing for results						
Results-oriented frameworks	B or A	C	B or A	C	B or A	C
Mutual accountability						
Mutual accountability	Yes	Yes	Yes	Yes	Yes	No

Note: Mongolia and Kyrgyzstan participated in both surveys conducted in 2006 and 2011. Tajikistan participated in the 2011 survey only, whereas Uzbekistan and Turkmenistan did not participate in the survey. Indicators below two scores of the target achievable are in Red and those achieved or near to achieving are in Green.

Source: OECD (81).

a Scored from A to D: A–highest, D–lowest.

b Rated on a scale of 1 (low) to 6 (high) in half-point increments (0.5). A score of 1 corresponds to a very weak performance and a score of 6 to a very strong performance.

c No target – Indicates that the indicator was not included in the previous monitoring survey in 2006.

All three countries have only 2–3 indicators for alignment and mutual accountability met out of 12 indicators for the Paris Declaration. As for the CAPS country health sectors, arguably these indicators would be slightly different in positive ways: ownership and alignment indicators for the health sector would show progress as preparation for and implementation of a health SWAp has improved MoH ownership and coordination capacity in Mongolia, Kyrgyzstan, and Tajikistan.

###  Aid coordination in health

We have extrapolated the current directions of aid coordination, observing a transition from ‘Donor coordination’ to ‘Development Partnerships’ as trade relationships progressively displace aid relationships, while still requiring a level of government coordination. Since the 4th High Level Forum on Aid Effectiveness in Busan (82), the understanding of aid coordination processes has broadened its scope beyond aid promoting country ownership and sustainability (82, 83). The implications of this paradigm change have been progressively reflected in the aid coordination of CAPS countries.

CAPS countries are located across the three evolutionary stages of aid coordination steps articulated by WHO (Table 5) in a framework for aid coordination. In this table adapted from WHO, we envisage a transition into a fourth stage as foreshadowed at Busan Forum.

**Table 5:** Aid coordination stages and their features

Stages	Characteristic	What is the expected outcome?	Dominant form of aid modalities	Countries
Stage one: Donor coordination	Main drive comes from the donors; government is passive as there are no systems to engage with the donors in policy dialogue.	Improved coordination of development partners	Donor-led projects	Uzbekistan-UNDP takes the lead role in aid coordination and EU is also involved in coordination of technical assistance programmes implemented jointly with other international partners (84) Turkmenistan – EU takes the lead role in aid coordination.
Stage two: Aid coordination	Increasing proactive engagement of government counterparts; establishment of aid coordination mechanisms	Improved aid effectiveness	Projects may still be dominant but better aligned with national priorities; initial SWAp steps could be taken; government increasingly takes the role for aid coordination	Tajikistan: government is increasingly aware of the importance of taking the lead role in aid coordination and is gradually taking the role formerly held by WHO (62).
Stage three: Development coordination	Government increasingly takes the initiative in policy dialogue; establishment of effective mechanisms of both government and external resources.	Improved development effectiveness; improved performance of the system	A SWAp; government-led aid coordination mechanism; possibly, budget support	Mongolia SWAp readiness exists, but without much progress since 2006; MoH has established a structure for coordinating external resources in accordance with its own priorities listed in the HSSMP. HSSMP mid-term review was undertaken using JANS. Kyrgyzstan: Kyrgyz MoH was the first of its kind to implement SWAp in former Soviet countries and demonstrated SWAps’ relevance and success to post-soviet contexts when applied appropriately (55, 66); National Health Reform Programme evaluation was undertaken using JANS.
Stage four: Development partnerships	Private/trade partners share roles and responsibilities.	Improved development effectiveness; trade relationships replace aid; improved governance (transparency and accountability).	Public–private partnership; there is no longer donor–recipient relationship; corporate sector's role in development still to be determined. (85).	None of the countries has established public–private partnership (PPP) in health. However, in other sectors, mostly in business sectors, public–private partnerships are under discussion in Mongolia and Kyrgyzstan.

Source: Adapted from WHO (62).

There is a lack of government-led aid coordination mechanisms in Turkmenistan and Uzbekistan. The UNDP takes the lead in an aid coordination role in Uzbekistan, and in Turkmenistan, the International Technical Assistance Coordination Unit under the Ministry of Finance is responsible for managing EU projects and programmes nationally (86).

Tajikistan, Kyrgyzstan, and Mongolia have had positive outcomes since 2005 in the coordination of donors and allocation of the aid flows (9, 12, 22). In Tajikistan and Mongolia, aid coordination units have been established within the MoH, initially led by the WHO, but progressively shifting to the MoH (54, 62). The most promising fact is that the government itself is actively involved to coordinate aid and promote the SWAps in these countries (54, 58), and contrasting with contexts where development partners have driven the SWAps process, undermining country ownership (87).

The development of a national health plan under government leadership has played an important role both in Kyrgyzstan and Mongolia in shifting aid from donor-driven aid projects to country-led programme support (55, 60) and in promoting capacity building amongst health policy makers (55, 58). With the development of their national health plan, Manas Taalimi 2006–2010, the Kyrgyz health sector has officially launched a SWAp greatly improving aid coordination (74).

As a result of a successful SWAp in Kyrgyzstan, the alignment and harmonisation with government policies in the health sector has been improved, and use of country systems have been increased from 3% in 2005 to 14% in 2007 (88); the health sector attracted more donor funds than ever (56). One of the key conditions set by external development agencies for disbursing funds within the SWAp framework was an annual increase of 0.6% in the state health budget as a percentage of total state expenditure. Total expenditure on health accounted for 6.4% of GDP in 2008, which meant that Kyrgyzstan was spending a higher share of GDP on health than many other countries of the former USSR (22). Supporting factors such as an inclusive policy process, a changing political environment and efforts to promote good governance were the key not only for success in the SWAP, but also in the overall health system, making the country a regional leader in the health system reform process (55).

In Mongolia, the largest health projects by ADB fully support the implementation of the Health Sector Strategic Master Plan (60). But a need for more effective coordination in CAPS countries still exists; aid coordination should guide donors towards health system's strengthening (60, 89). Early public–private partnerships (PPP) are emerging in Mongolia and Kyrgyzstan, although they are in a very early stage of maturity, and an appropriate legal framework and institutions to attract partners in PPP is needed (90, 91).

## Conclusions

Despite commonalities at the start of the post-Soviet era, CAPS countries developed two distinctive trajectories due to their differing politics and governance. Kyrgyzstan, Mongolia, and Tajikistan have demonstrated better prospects for effective development cooperation and improved health outcomes. However, in Uzbekistan and Turkmenistan, their control-oriented management culture and opaque management processes, appear to have discouraged the engagement of effective development partners, and as a result, have hindered development cooperation.

The influence of donors, both financially and technically, remains crucial to health sector reform despite their relatively small contribution to overall health budgets. As a result of the existing health infrastructure and human resources established under the Soviet system, the CAPS countries have had the potential to achieve better health outcomes in a relatively short time, if the right reform processes are undertaken under the right leadership. This will also need the engagement of political elites and partners to improve issues beyond the health sector. The studies of CAPS countries suggest that regardless of current economic status, resistance to developing more open and accountable relationships with the donors can result in systems stagnation, and slow progress to improve health and socio-economic indices. Factors that influence aid effectiveness such as control or corruption, voice and accountability have not shown much progress in the past 10 years, which had been reflected in the slow progress in Paris Declaration indicators. The key lessons from our analysis of the development of aid relationships in health in CAPS countries are that:

1. *New aid relationships could offer new opportunities for both donors and recipients*. Neither governments nor donors had any experience of working together in CAPS countries prior to the collapse of the Soviet Union. The positive outcomes for Tajikistan, Kyrgyzstan, and Mongolia who were quite receptive to the new relationships, have been significant, compared to the less open and transparent Uzbekistan and Turkmenistan.
2. *Fewer partners do not necessarily mean less fragmentation*. Even where there have been limited numbers of donors, overlap and duplication of the projects being implemented, and their parallel management mechanisms, have resulted in administrative and capacity burdens to local systems. With health system reform processes in these countries requiring a paradigm shift at every level of the system, donor coordination is critical, regardless of the number of donors.
3. *Aid modalities chosen must reinforce ownership and sustainability*. While the diversity of donors, policies, and approaches makes it difficult to preference any single aid modality, using local systems, local management, and governance is a key to sustainable development. Where project aid continues, it needs to be aligned with national priorities, and effective coordination by the government is critical to ensuring its contribution to health systems strengthening. Country-led capacity building processes also remain crucial, as was evident in the health sector plan development in Tajikistan, Kyrgyzstan, and Mongolia.
4. *Aid coordination beyond the health sector is needed to bring real development effectiveness for health*. Many factors outside health affect development effectiveness: the broader political context, governance, management culture and capacity indirectly affect health outcomes and systems development. Development effectiveness requires its own time for in-country capacity building, and the lack of absorptive capacity remains a challenge, especially in contexts such as Mongolia and Turkmenistan, where economic growth has occurred in a relatively short time. In these contexts, capacity shortage, if not resource shortage, remains as an issue, necessitating continued technical assistance.

## Appendix

**Annex 1:** Representation of the key informants

	Experiences in		
	
Parent Institution	Kyrgyzstan	Mongolia	Uzbekistan
Ministry of Health		1	
Asian Development Bank	1	2	1
UNICEF	1	1	1
GIZ	1	1	
District Health Centre		1	
Total	3	6	2

**Annex 2:** Abbreviations used in Table 2

ADB	Asian Development Bank
AusAid	Australian Agency for International Development
CIDA	Canadian International Development Agency
DANIDA	Danish International Development Agency
DFID	Department for International Development (UK)
GAVI	Global Alliance for Vaccines and Immunisation
GTZ	Gesellschaft für Technische Zusammenarbeit (German Technical Development Agency, now renamed as GIZ)
JICA	Japan International Cooperation Agency
MSF	Médecins sans Frontières (Doctors without Borders)
NLM	Norwegian Lutheran Mission
SDC	Swiss Agency for Development and Cooperation
SIDA	Swedish International Development Cooperation Agency
UNAIDS	Joint United Nations Programme on HIV/AIDS
UNFPA	United Nations Population Fund
UNICEF	United Nations Children's Fund
USAID	United States Agency for International Development
WHO	World Health Organization
